# Adenosine Inhibits the Release of Arachidonic Acid in Activated Human Peripheral Mononuclear Cells. A Proposed Model for Physiologic and Pathologic Regulation in Systemic Lupus Erythematosus

**DOI:** 10.1100/tsw.2011.88

**Published:** 2011-04-19

**Authors:** Sándor Sipka

**Affiliations:** 3rd Department of Internal Medicine, University of Debrecen, Debrecen, Hungary

**Keywords:** adenosine, arachidonic, acid, phopholipase A_2_, protein kinase C, systemic lupus erythematosus (SLE)

## Abstract

In the current work, the pathways are presented and reviewed showing how adenosine acts on the production and release of arachidonic acid (AA) in activated human monocytes by the involvement of various phospholipase A_2_ (PLA_2_) and protein kinase C (PKC) enzymes in physiological (normal) conditions and in a pathologic state in systemic lupus erythematosus (SLE). Two molecules of activated monocytes mainly determine the actual amounts of AA released: (1) interleukin-1β (IL-1β) increasing and (2) adenosine (Ado) suppressing this process. The AA production of monocytes mainly depends on two (IV and VI) types of PLA_2_ enzymes. PKCα phosphorylates the cytosolic, Ca-dependent and steroid-sensitive PLA_2_ (type IV), whereas PKCδ phosphorylates the Ca-independent PLA_2_ (type VI). By the suppression of IL-1β production in the activated human monocytes, adenosine can decrease the release of AA causing a diminished phosphorylation of both PKC isoenzymes. In SLE monocytes, the disease-specific decreased release of AA that we found earlier could be related to the decreased expression of PKCδ. These pathways are summarized in a proposed model.

## PHYSIOLOGICAL ROLE OF ADENOSINE

Adenosine (Ado) is one of the vital molecules of biological evolution. As a nucleoside component and derivative of adenosine triphosphate (ATP), Ado itself, as an individual molecule, has a wide range of activities in the nervous, reproductive, cardiac, renal, hepatic, and respiratory systems[1] and in the inhibition of inflammation[2]. Ado as a metabolite of ATP has a very short half-life (1.5 sec) due to its rapid metabolism. It accumulates in the area where ATP is utilized, but not reformed, e.g., during ischemia and sepsis[3,4]. Unlike ATP, Ado exists free in the cytosol of all cells, and is transported in and out of the cells by a membrane transporter system. It is not a conventional transmitter, but a sort of local hormone or a "homeostatic modulator"[3].

Following its release, Ado binds to extracellularly expressed adenosine receptors, which belong to the family of G protein–coupled receptors. There are four different types of adenosine receptors (adenosine $A_1$, $A_{2A}$, $A_{2B}$, and $A_3$). The action of Ado is determined by the type of receptor to which it binds[5]. Although Ado is constitutively present in biologic systems extracellularly at a very low concentration (<1 μM), its concentration increases under metabolically stressed conditions like acute tissue injury or sepsis[3,6]. Ado is an endogenous regulator of innate immunity[7]. In addition, Ado produced by $CD4^+CD25^+$ Treg cells contributes to the resolution of inflammation and the regulation of the immune/inflammatory cell phenotype[8].

Under normal conditions, Ado is mainly generated at the intracellular level from 5-adenosylhomocysteine by the action of S-adenosylhomocysteine hydrolase[9]. During acute inflammation or hypoxia, ATP is dephosphorylated to Ado via 5'-nucleotidase enzymes (CD73, endo-5' nucleotidase and CD39, ecto-5' nucleotidase). Adenosine kinase–mediated salvage activity is also suppressed and leads to further increase in Ado levels[6]. Once Ado is formed, its extracellular level is controlled by two types of purine or nucleoside transporters: (1) transporting inside or outside the cell, depending on the concentration (ENT1); (2) facilitating the intracellular influx of Ado against the concentration gradients (ENT2)[10].

Ado bioavailability is also influenced by adenosine deaminase (ADA) catabolizing Ado into inosine, also a weak agonist for $A_1$ receptors in a higher concentration range (>100 μM) or an immunomodulatory molecule for $A_{2A}$ and $A_3$ receptors. ADA plays a crucial role in the regulation of the extracellular or plasma levels of Ado, e.g., human monocytes in activated state secrete this enzyme[11].

Ado acts as a sensor and provides information to the immune system about tissue damage or acute inflammatory changes.

## PATHOPHYSIOLOGICAL AND PHARMACOLOGICAL ROLES OF ADO

The pathologic role of Ado was recognized in bronchial asthma (as a bronchoconstrictor), refractory primary pulmonary hypertension (causing pulmonary vasodilation), inflammatory bowel disease, anesthesia, epilepsy, ischemia/reperfusion injury, sepsis (decrease in superoxide anion release, increase in IL-10), Parkinson's disease (benefit of $A_{2A}$ receptor antagonists), paroxismal cardiac tachycardia (benefit of Ado infusion), and hypertriglyceridemia (Ado normalizes the triglyceride level)[3].

## PHYSIOLOGICAL ROLE OF ARACHIDONIC ACID

Arachidonic acid (AA) is a polyunsaturated omega-6 fatty acid 20:4 (ω-6). AA is present in the phospholipids (especially in phosphatidylethanolamine, phosphatidylcholine, and phosphatidylinositides) of membranes of the body's cells, and is abundant in the brain and muscles.

AA is involved in cellular signaling as a lipid second messenger involved in the regulation of signaling enzymes, such as PLC-γ, PLC-δ, and protein kinase C (PKC) α and γ isoforms. AA is a key mediator of inflammation.

AA is freed from the phopholipid molecule by the enzyme phospholipase $A_2$ ($PLA_2$), which cleaves off the fatty acid, but can also be generated from diacyl glycerol (DAG) by DAG lipase. AA generated for signaling purposes appears to be delivered by the action of a phosphatidylcholine-specific $Ca^{2+}$-dependent cytosolic $PLA_2$ (type IV $cPLA_2$, 85 kDa), whereas inflammatory AA is generated by the action of a low-molecular-weight secretory $PLA_2$ (type II $sPLA_2$, 14–18 kDa) and the $Ca^{2+}$-independent cytosolic $PLA_2$ (type VI, $iPLA_2$).

AA is a precursor in the production of eicosanoids:

- The enzymes cyclooxygenase and peroxidase lead to prostaglandin $H_2$, which in turn is used to produce the prostaglandins, prostacyclin, and thromboxanes.
- The enzyme 5-lipoxygenase leads to 5-HPETE, which in turn is used to produce the leukotrienes.
- AA is also used in the biosynthesis of anandamine.
- Some AA is converted into hydroxyeicosatetraenoic acid (HETEs) and epoxyeicosatrienoic acids (EETs) by epoxygenase[12,13].

Whereas AA is one of the main activators of NADPH oxidase producing superoxide anion in the phagocytes (monocytes, neutrophils), prostaglandin $E_2$ has potent immunosuppressive and anti-inflammatory effects[14,15].

## ADO DOSE DEPENDENTLY CAN INHIBIT THE RELEASE OF AA IN HUMAN MONONUCLEAR CELLS (MONOCYTES, LYMPHOCYTES) ACTIVATED BY PHORBOL ESTER AND $CA^{2+}$ IONOPHORE

Testing the peripheral mononuclear cells (monocytes and lymphocytes) activated by phorbol ester and $CA^{2+}$ ionophore (A-23187) for 4 h in RPMI-1640 medium completed with 10% fetal calf serum (80 μg/ml gentamycin and 2 mM glutamine in cell culture milieu, and ADA inhibitor EHNA), we found earlier[16] that Ado significantly could inhibit the release of $^3$H-AA in the concentration range higher than $10^{-6}$ M. Using the Ado receptor–specific agonist ligands, $A_1$-specific CPA and $A_2$-specific CGS 21680, the significant inhibitions were found at a much lower dose, at $10^{-7}$ M. The triggering of the $A_{2B}$ and $A_3$ receptors did not result in a detectable change. These data are shown in Fig. 1.

The significant moderation of the $A_1$ and $A_2$ receptor-mediated inhibition of AA release could be achieved by the preincubation with the specific antagonists molecules (DPCPX for CPA, ZM2431850 for CGS 21680) presented in Fig. 2[16].

In this experimental model of peripheral mononuclear cells, the important elements were as follows: (1) the activating agents (phorbol ester and the $Ca^{2+}$ ionophore) could trigger both the monocytes producing AA and some cytokines (first of all IL-1β); and (2) the lymphocytes producing mostly proinflammatory cytokines such as tumor necrosis factor (TNF)α or interferon (IFN)γ. The role of IL-1β was crucial because the monocytes required the presence of IL-1β to produce AA continuously[17,18]. In addition, the proinflammatory cytokines derived from the activated lymphocytes could further activate the monocytes to produce both IL-1β and AA in a self-perpetuating positive feedback regulatory pathway. It was known already that the $A_{2A}$ receptor agonists could inhibit the release of proinflammatory IL-2, TNFα, and IFNγ from activated human T cells[19]. Our new observation was that Ado could enter this cycle by the potent inhibition of IL-1β release in the activated monocytes[20]. When the extracellular level of IL-1β was decreased, the release of AA was also diminished.

In human neutrophil granulocytes, it was observed that Ado was a natural endogenous suppressor of AA release and leukotriene biosynthesis by the inhibition of the $cPLA_2$ via the $A_{2A}$ receptors[21]. Ado accumulated in the leukocyte suspensions as a consequence of the extracellular breakdown of ATP[22] and its concentrations exerted suppressive effects on the neutrophil actions[23].

It is important to mention that in mature T lymphocytes, the release of AA is very low because they do not have $cPLA_2$[24]. Thus, the monocytes were the sources of AA release from the suspensions of activated mononuclear cells (lymphocyte + monocytes) in our system.

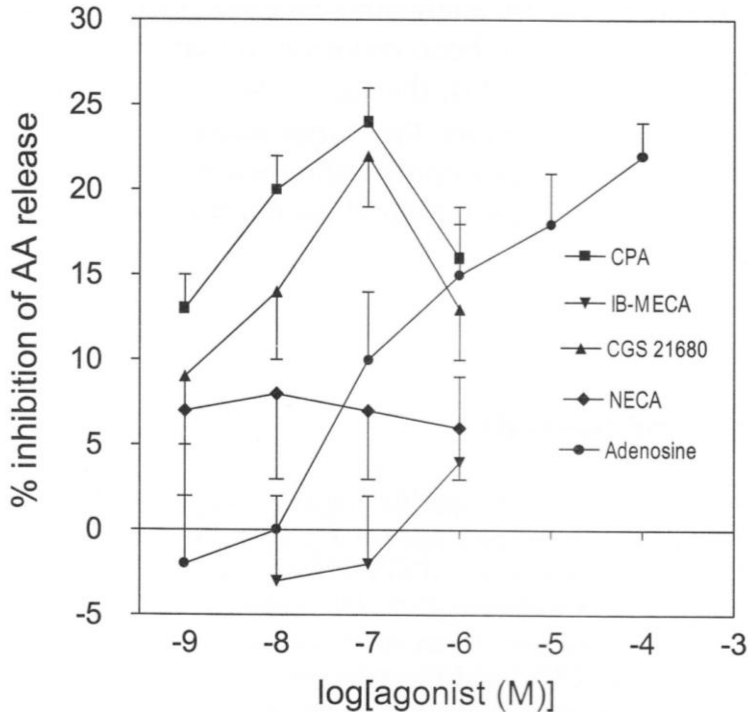

**FIGURE 1.** Effect of Ado and subtype-specific Ado receptor activators on the release of AA from human peripheral mononuclear cells activated by phorbol ester (PMA) and $Ca^{2+}$ ionophore (A-23187). Actions of Ado and the subtype-specific Ado receptor agonists (CPA: $A_1$, CGS 21680: $A_{2A}$, IB-MECA: $A_3$, NECA: $A_2$ receptor activators) on the AA production by activated human peripheral mononuclear cells (monocytes) were studied in the presence of 10 μM of the Ado inhibitor EHNA (erythro-9-[2-hydroxy-3-nonyl] adenine hydrochloride). In order to get an $A_{2B}$ effect, NECA was administered in the presence of $A_1$, $A_{2A}$, and $A_3$ receptor antagonists (0.3 μM DPCPX, 0.1 μM ZM 243185, and 10 nM MRS 1191, respectively). The mean PMA+A23187 induced and released $^3H$ AA activity was 2855.4 ± 239 S.E.M. dpm before the exposition to Ado and the Ado receptor agonists. Values are expressed as the mean ± S.E.M. of five to seven experiments. *Abbreviations:* AA, arachidonic acid; CPA, $N^6$-cyclopentyladenosine ($A_1$ receptor agonist); CGS 21680, 2-p-(2-carboxyethyl)-amino-5-N-ethylcarboxiamido-adenosine ($A_{2A}$ receptor agonist); NECA, 5-N-ethylcarboxamidoadenosine (nonspecific $A_2$ receptor agonist); IB-MECA, N6-(3-iodobenzyl)-5(N-methylcarboxamidoadenosine) ($A_3$ receptor agonist).

## THE ROLE OF PKCδ IN THE IMPAIRED RELEASE OF AA FROM THE MONOCYTES OF PATIENTS WITH SYSTEMIC LUPUS ERYTHEMATOSUS

In 2001, we found that the monocytes of systemic lupus erythematosus (SLE) patients without corticosteroid treatment, stimulated by the PKC activating molecule, phorbol ester, could produce significantly less AA than those of healthy controls, suggesting a defect in the PKC enzyme system of these patients. On the other hand, in the patients taking higher doses of steroid (16–32 mg/day), the release of AA was not decreased any more because the activity of $iPLA_2$ was normalized[25]. In this case, the steroid treatment could elevate the decreased expression of PKCδ in the monocytes, and this enzyme could phosphorylate $iPLA_2$ to produce AA[27].

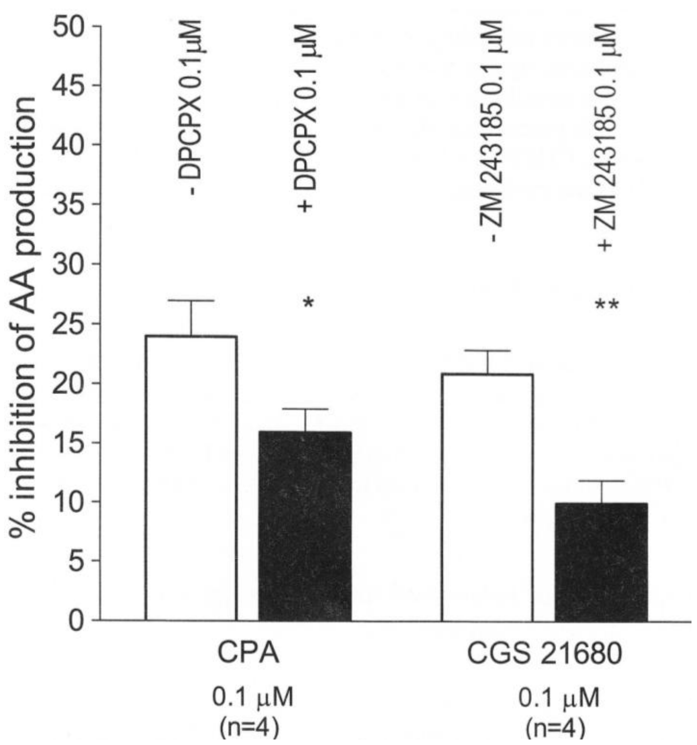

**FIGURE 2.** Effects of specific $A_1$ (DPCPX) and $A_{2A}$ (ZM 243185) antagonists on the inhibition of AA release induced by subtype-selective Ado receptor agonists in activated human peripheral mononuclear cells (monocytes). Values are expressed as the mean ± S.E.M. of 4-4 experiments. *$p < 0.05$; **$p < 0.01$. *Abbreviations:* AA, arachidonic acid; CPA, $N^6$-cyclopentyladenosine ($A_1$ receptor agonist); CGS 21680, 2-p-[2-carboxyethyl]-amino-5-N-ethylcarboxiamido-adenosine ($A_2$ receptor agonist); DPCPX: 8-cyclophenyl-1.3-dipropylxanthin ($A_1$ receptor antagonist); ZM243185, 4-2(-[7amino-2-(furyl)[1,2,4]triazolo[2,3,][1,3,5]triazin-5-ylamino]ethyl) phenol ($A_{2A}$ receptor antagonist).

Furthermore, later (in 2004) we observed a SLE-specific decreased expression of PKC isoenzymes, both in the monocytes and T lymphocytes. In the monocytes, the expression of PKCε and δ; in the T cells, the expression of PKCβ, ε, δ, η, θ, and ξ, were significantly decreased compared to the cells of healthy controls. The treatment of the SLE cells with hydrocortisone *in vitro* could restore these defects of isoenzyme expression in both cell types with the exception of PKCθ in the lymphocytes[26]. In addition, by the transfection of HaCaT cells with the cDNA of the various human PKC isoenzymes, we found that the expectable increased production of AA occurred only in the cells overexpressing PKCβ and δ. Thus, we concluded that the decreased release of AA in the monocytes of SLE patients could only be explained by the decreased expression of PKCδ, because the expression of PKCβ was not impaired in these cells[27].

The pathomechanism of acute inflammation, “flare”, in SLE is mostly related to immunocomplexes, and mostly represents the type III form of immunological reactions, where the tissue destruction is caused by lysosomal enzymes or cytokines. From the derivatives of AA, released in a less amount from the monocytes of SLE patients, possibly the decreased production of $PGE_2$ can still contribute to the acute symptoms of disease, as its suppressor effect on the T cells becomes limited[28]. It is also known that, in

SLE, not only is the number reduced, but also the suppressor function of the $CD4^+CD25^+$ FOX $P3^+$ Treg cells producing Ado is impaired[29,30].

## A PROPOSED MODEL FOR THE REGULATION OF AA RELEASE BY ADO IN THE MONOCYTES FROM HEALTHY SUBJECTS AND SLE PATIENTS

The actual production and release of AA from activated human monocytes is mainly regulated as follows: (1) the group of *$PLA_2$ enzymes* consisting of the $Ca^{2+}$-dependent, steroid-sensitive, cytoplasmic, type IV $PLA_2$ and $Ca^{2+}$-indpendent, steroid nonsensitive, type VI $PLA_2$, both produce AA; (2) *IL-1β*, the cytokine product of activated monocytes is required for the phosphorylation of both PKCα (phosphorylating $cPLA_2$) and PKCδ (phosphorylating $iPLA_2$)[31] to produce and release AA; thus, IL-1β is also a key component of the positive feedback regulation loop of monocyte activation; (3) *Ado* derived from both the activated monocytes and the suppressor Treg cells can inhibit the production and release of IL-1β, leading to the suppressed production of AA and to the moderation of inflammation; (4) *ADA* derived from the activated monocytes can destroy Ado[11]; (5) *IL-1 receptor antagonist (IL-1Ra)* is also produced by the activated monocytes and can limit the action of IL-1β[32]; (6) AA can activate *NADPH oxidase* in the monocytes to produce superoxide radicals, partly catalyzed to hydrogen peroxide by superoxide dismutase; and both ROS molecules can further activate the monocytes leading to an increased mobilization of AA[33]. Ado can block the PKCα-mediated release of AA via the $A_1$ and $A_{2A}$ receptors[34,35], whereas it does not seem to have any effect on PKCδ ($A_{2B}$ and $A_3$ receptor)–mediated AA production[34], as we also observed. The proposed model is presented in Fig. 3.

In the SLE monocytes, the decreased expression of PKCδ[16,17] required for the phosphorylation of $iPLA_2$[16] may play the crucial role in the defected production and release of AA. This lack of AA also causes the decreased production of the immunosuppressive $PGE_2$[28,30]. In addition, the reduced production of IL-1β[32] and the increased release of IL1-Ra[11] also can contribute to the impaired AA metabolism. Clinical observations showing the decreased number of $CD4^+CD25^+$ Treg cells producing Ado[29,30] and the increased release of ADA[11,36,37] suggest that the serum level of Ado used to be lower in the SLE patients than in the healthy controls. However, the serum level of AA strongly depends on the dose of glucocorticosteroids used in the therapy because these drugs not only restore the impaired (decreased) AA production in the lupus patients, but they can result in even significant elevations[25,38].

## CONCLUSIONS

(1) The main molecules regulating the actual production and release of AA in the activated human monocytes can be classified into two groups: stimulating molecules ($PLA_2$ type IV [PKCα], $PLA_2$ type VI [PKCδ], IL-1β, AA, ADA, superoxide anion, hydrogen peroxide) and inhibiting molecules (Ado, IL-1Ra, $PGE_2$). (2) In the decreased production of AA by the monocytes of SLE patients, the decreased expression of PKCδ, the suppressed production of IL-1β, and the increased release and serum level of IL-1Ra can play the main roles.

## ACKNOWLEDGMENT

I am thankful to all my coworkers for taking part in the works related to this article. This work was supported by the grant OTKA 71883.

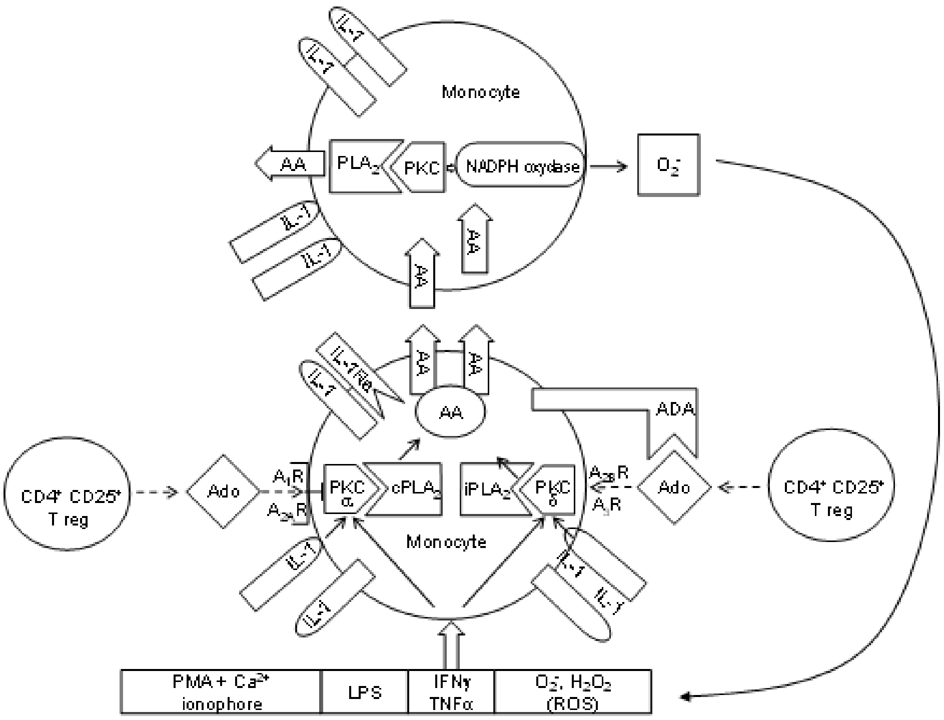

**FIGURE 3.** A proposed model for the regulation of AA release by Ado in the monocytes of healthy subjects. *Abbreviations:* Ado, adenosine; AR, adenosine receptor; ADA, adenosine deaminase; AA, arachidonic acid; $PLA_2$, phospholipase $A_2$; $cPLA_2$, cytoplasmic $PLA_2$; $iPLA_2$, calcium-independent $PLA_2$; PKC, protein kinase C; IL-1, interleukin 1β; IL-1Ra, interleukin-1 receptor antagonist; IFNγ, interferon γ; LPS, *Escherichia coli* lipopolysaccharide; PMA, phorbol myristate acetate; ROS, reactive oxygen species.
